# Vitamin D status in cats with feline immunodeficiency virus

**DOI:** 10.1002/vms3.11

**Published:** 2015-11-23

**Authors:** Helen F. Titmarsh, Stephanie M. Lalor, Severine Tasker, Emily N. Barker, Jacqueline Berry, Danielle Gunn‐More, Richard J Mellanby

**Affiliations:** ^1^ The Royal (Dick) School of Veterinary Studies and the Roslin Institute University of Edinburgh Roslin Edinburgh UK; ^2^ School of Veterinary Sciences University of Bristol Langford Bristol UK; ^3^ Vitamin D Research Laboratory Department of Medicine Manchester Royal Infirmary Manchester UK

**Keywords:** vitamin D, feline, feline immunodeficiency virus, 25(OH)D, human immunodeficiency virus

## Abstract

Feline immunodeficiency virus (FIV) is a lentivirus that can lead to a syndrome of acquired immune dysfunction. Infected cats often remain asymptomatic for several years before immune dysfunction leads to an increased risk for the development of systemic diseases, neoplasia and opportunistic infections. FIV is structurally related to human immunodeficiency virus (HIV) and the pathogenesis of FIV‐related disease is similar to that seen in HIV‐infected patients. Observational studies have documented an association between low plasma vitamin D and HIV infection. Vitamin D status has been shown to be associated with HIV‐related disease progression, morbidity and mortality. The objective of this study was to examine the hypothesis that vitamin D status, as assessed by serum 25‐hydroxyvitamin D [25(OH)D] concentrations, are lower in cats with FIV infection compared to healthy control cats. Serum 25(OH)D concentrations were measured in 20 healthy cats, 39 hospitalized ill cats and 59 cats infected with FIV. Cats which were FIV infected had significantly lower 25(OH)D concentrations compared to healthy control cats. Serum 25(OH)D concentrations were not significantly different between FIV‐infected cats and hospitalized ill cats. Further investigations are warranted to determine whether vitamin D status influences the prognosis of cats infected with FIV.

## Introduction

Feline immunodeficiency virus (FIV) is a retrovirus of domestic cats and other Felidae which belongs to the lentivirus genus (Pedersen *et al*. 1989). Transmission primarily occurs by horizontal spread between adult cats through bites wounds. Risk factors for infection include cats with outdoor access and cats with aggressive behaviour (Hartmann 2011). Once FIV infection has been acquired most cats experience a transient period of viraemia accompanied by pyrexia, lymphadenopathy, lethargy, anorexia and leukopenia (Yamamoto *et al*. 1988; Pedersen *et al*. 1989; Hartmann 2011). After several weeks the viraemia subsides and cats often enter a prolonged asymptomatic phase infection lasting many years. During this clinically asymptomatic phase there is a progressive decline in CD4+ T‐lymphocytes (Dunham & Jarrett, 2006). Some infected cats will remain asymptomatic despite this decline in CD4+ T‐cells; however, in other cats the decline in CD4+ T‐cells and resulting immune dysfunction leads to an increased susceptibility to infections, immune‐mediated diseases and neoplasia (Hartmann 2011).

FIV shares many morphological and pathophysiological features with the human lentivirus, human immunodeficiency virus (HIV). Like HIV, FIV is characterized by tropism for lymphocytes, macrophages and cells of the central nervous system (Dow *et al*. 1990; English *et al*. 1993). In the host species, infection with each virus leads to a progressive acquired immunodeficiency syndrome. The immune dysfunction seen as a result of HIV and FIV infections includes cytokine dysregulation, inappropriate activation of immune regulatory cells and T‐cell anergy and apoptosis (Tompkins & Tompkins 2008). Overall, these conditions are associated with a pro‐inflammatory state and an increase in plasma cell activity with non‐specific immunoglobulin production.

A large number of studies have investigated the relationship between serum vitamin D levels, as assessed by serum 25‐hydroxyvitamin D [25(OH)D] concentrations, and the incidence, progression and morbidity associated with HIV infection. These studies have documented a high incidence of low vitamin D status in patients infected with HIV (Villamor 2006; Lake & Adams 2011). Sub‐optimal vitamin D status and low serum concentrations of the active form of vitamin D, calcitriol have been associated with lower CD4+ T‐cell counts, HIV progression and AIDs‐related morbidity and survival (Haug *et al*. 1994; Mehta *et al*. 2011; Vescini *et al*. 2011; Kim *et al*. 2012). One large‐scale study has also identified vitamin D deficiency as an independent risk factor for all‐cause mortality in HIV‐positive patients (Viard, Jean‐Paul, *et al*. 2011).

The mechanistic link between vitamin D deficiency and HIV progression and AIDS related morbidity and survival may be via the marked immunomodulatory properties of biologically active vitamin D, 1,25 dihydroxycholecalciferol (1,25(OH)_2_D). The roles of calcitriol within the immune system include; down‐regulation of pro‐inflammatory cytokines, suppression of T‐cell activation, shifting of T‐cell responses from a T_H_1 to T_H_2 type response, regulation of monocyte chemotaxis, macrophage function and dendritic cell phenotypes, and regulation of the production of antimicrobial and antiviral peptides (Jeremy *et al*. 2011; Prietl *et al*. 2013). Given the known immunomodulatory function of vitamin D metabolites, it is possible that a combination of HIV infection and insufficient vitamin D could be additive in promoting immune dysfunction in patients infected with HIV. Due to the increasing evidence of the immunomodulatory effects of vitamin D and the association between sub‐normal vitamin D levels and increased mortality, there is growing interest in the benefit of vitamin D supplementation in HIV‐positive patients (Villamor 2006).

Given the similarities between HIV and FIV infection, we hypothesized that the vitamin D status of FIV‐infected cats would be significantly lower compared to healthy control cats. The aim of this retrospective study was to measure serum 25(OH)D concentrations in cats infected with FIV, healthy cats and a general population of hospitalized ill cats. Although it is possible to measure a number of vitamin D metabolites, vitamin D status is typically only assessed by measuring 25(OH)D concentrations (Holick 2009). Although calcitriol is the active form of vitamin D, assessing calcitriol concentrations does not provide accurate information regarding vitamin D sufficiency or deficiency. Unlike calcitriol production, the hepatic production of 25(OH)D is not actively regulated, meaning that serum 25(OH)D directly reflects either dietary vitamin D intake or cutaneous synthesis (Holick 2009). The half‐life of 25(OH)D is life of approximately 2–3 weeks reflecting vitamin D intake and synthesis over this period of time (Holick 2009). Therefore, 25(OH)D concentrations provide the most accurate assessment of vitamin D status (Holick 2009).

## Material and methods

### Study population

The study was undertaken using residual serum samples from cats treated at the Hospital for Small Animals, Royal Dick School for Veterinary Studies, University of Edinburgh and clinical samples submitted to Langford Veterinary Services, University of Bristol for determination of FIV status.

Healthy control cats were defined as healthy based on history, clinical examination findings, routine haematology and serum biochemistry. Cats enrolled in this group were blood sampled for the primary clinical purposes of assessment of PCV, biochemistry, FIV status and feline leukaemia virus (FeLV) status prior to blood donation or for routine FIV/FeLV testing prior to re‐homing. FIV and FeLV status was tested using enzyme‐linked immunosorbent assay (ELISA) (SNAP^®^ FIV/FeLV Combo Test; IDEXX Corp., Portland, ME, USA).

The hospitalized ill cats were consecutively recruited from cats admitted to Hospital for Small Animals, Royal Dick School for Veterinary Studies, University of Edinburgh. Cats were included in this group if they had a history and clinical examination findings indicating that diagnostic interventions were warranted. Signalment, history, recent drug administration, haematology results, serum biochemistry results and final diagnosis were recorded for each case. FIV status was tested using an in house ELISA test (SNAP^®^ FIV/FeLV Combo Test; or CITE Combo FIV‐FeLV, Agritech Systems, Portland, ME, USA; or Speed^®^ FIV, Virbac Animal Health, La Seyne sur Mer, France). Cats which tested positive for FIV were excluded from the hospitalized sick group. Cats that had been treated with corticosteroids in the 2 weeks prior to sample collection were also excluded. This is because glucocorticoids have been linked to disturbances in calcium metabolism in humans and companion animals (Hahn *et al*. 1981; Ramsey *et al*. 2005).

Samples retrieved for inclusion in the FIV‐infected group were obtained from residual samples submitted to Langford Veterinary Services, University of Bristol to establish FIV status. Each of the cats recruited tested positive for FIV by ELISA (PetCheck^®^ FIV antigen ELISA, IDEXX Corp.)

### Vitamin D measurement

Following handling of blood samples for routine diagnostic procedures, serum samples were stored at −20°C until 25(OH)D levels were measured. Samples were sent frozen in batches and 25(OH)D levels measured by high‐performance liquid chromatography (HPLC). Briefly, samples were extracted using acetonitrile and applied to C18 Silica Sep‐paks (Waters Associates, Milford, MA, USA). Separation of metabolites was by straight phase HPLC (Waters Associates) using a Hewlett‐Packard Zorbax‐Sil Column (Hichrom, Reading, UK) eluted with hexane:propan‐2‐ol:methanol (92:4:4). Serum concentrations of the two major vitamers of 25(OH)D, 25(OH)D_2_ (ergocalciferol) and 25(OH)D_3_ (cholecalciferol), were measured separately by application to a second Zorbax‐Sil Column eluted with hexane:propan‐2‐ol (98:2) and quantified by UV absorbance at 265 nm and corrected for recovery (sensitivity 5 nmol/L, intra‐ and inter‐assay coefficients of variation 3.0% and 4.2%, respectively). Vitamer concentrations were combined and results expressed as total 25(OH)D as described previously (Mawer *et al*. 1990). The assay laboratory is accredited to ISO 9001:2008 and ISO 13485:2003.

### Statistical analyses

Serum 25(OH)D concentrations were compared between healthy cats, hospitalized ill cats and FIV‐infected cats using the Kruskal–Wallis test with post‐test Dunn's multiple comparison tests. Statistical analysis was performed with the commercial software package GraphPad Prism 6 (Graphpad Software, La Jolia, CA, USA). *P *<* *0.05 was considered significant. The study was approved by The Royal Dick School of Veterinary Studies Ethical Review Committee.

## Results

### Signalment

The 20 cats in the healthy control group comprised 13 domestic short‐haired, 2 domestic long‐haired, 2 British short hairs, 2 Maine Coon cats and 1 Bengal cat. Eleven were neutered males, 6 were neutered females, two were entire males and one was an entire female. The ages of the cats in the healthy control group ranged from 2 to 11 years, with a median age of 5.5 years.

The 39 cats in the hospitalized ill group comprised 26 domestic short‐haired, 5 domestic long‐haired, 3 Maine Coon, 2 Bengal, 1 Burmese, 1 Norwegian Forest Cat and 1 Ragdoll cats. Twenty‐four cats were neutered males, 2 were male entire, and 13 were neutered females. Their ages ranged between 4 months and 18 years, with a median age of 8 years. The cats were diagnosed with a range of medical conditions including: aortic thromboembolism (1), biliary carcinoma (1) chronic kidney disease (3), congestive heart failure (2), diabetes mellitus (1), dysautonomia (1), feline infectious peritonitis (1) fibrosarcoma (1), idiopathic chylothorax (1), idiopathic feline lower urinary tract disease (3), idiopathic megaoesophagus (1) hepatic disease (1), herpes virus (1), hyperthyroidism (7), inflammatory bowel disease (2), gastrointestinal lymphoma (2), renal lymphoma (1), multicentric lymphoma (1), lymphadenopathy (1), myelodysplasia (1), oral squamous cell carcinoma (1), pyothorax (3), stomatitis (1) and urolithiasis (1).

There was no clinical data available for the 59 cats in the FIV‐infected group. The samples used were the stored serum sample from known FIV positive cats. Clinical data was not available for archived FIV positive samples.

### Serum 25(OH)D concentrations

The median serum 25(OH)D concentration in the healthy cats was 44.7 ng/mL (range 14.9–61.0 ng/mL). For the hospitalized ill cats, the median 25(OH)D concentration was 30.9 ng/mL (range 7.1–82.40 ng/mL). FIV‐infected cats had a median 25(OH)D concentration of 32.10 ng/mL (range 5.0–62.4 ng/mL).

**Figure 1:**
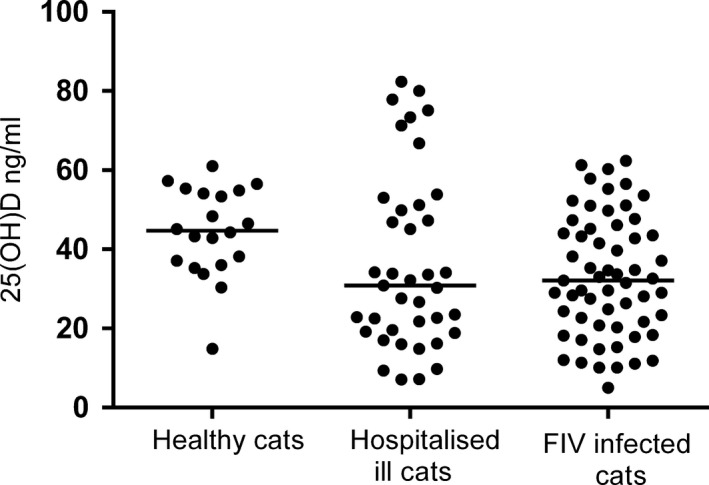
Serum 25(OH)D concentrations in healthy cats, hospitalized ill cats and FIV‐infected cats. 25(OH)D, 25‐hydroxyvitamin D; FIV, Feline immunodeficiency virus.

Serum 25(OH)D concentrations were significantly different between healthy control cats, hospitalized ill cats and FIV‐infected cats (*P* < 0.05); (Fig. 1). There was a significant difference in 25(OH)D concentrations between healthy cats and FIV‐infected cats (*P* < 0.05) and between healthy cats and hospitalized sick cats (*P* < 0.05). There was no difference in the serum 25(OH)D concentrations between the hospitalized ill cats and the FIV‐infected cats.

## Discussion

The central finding of this study is that FIV‐infected cats have significantly lower serum vitamin D concentrations than healthy cats. This observation is similar to the findings of numerous studies in humans which have documented lower serum levels of vitamin D in HIV‐positive patients (Lake & Adams 2011). Despite the well‐documented association, the clinical relevancy of the low vitamin D status in patients with HIV is not fully understood. Since HIV is a disease of immune dysfunction and calcitriol is known to have immunomodulatory properties, several studies have investigated whether vitamin D influences the immune response of HIV‐positive patients. As an intracellular virus, HIV replicates and evades the host‐immune response by down regulating cell‐mediated immune responses, (Peterlin & Trono 2003). Physiological doses of calcitriol have been shown to trigger autophagy and therefore inhibit HIV replication (Campbell & Spector 2011). Furthermore the addition of vitamin D antagonists decreases the inhibition of HIV‐1 replication (Campbell & Spector 2011). In addition calcitriol pre‐treatment has also been shown in vitro to decrease HIV infection by suppressing viral replication, (Connor *et al* 1991) to increase monocyte chemotaxis (Girasole *et al*. 1990) and to improve the monocyte maturation defects seen in HIV‐positive patient (Haug, Muller *et al*. 1996). Calictriol has also been shown to decrease the number of *Mycobacterium avium* bacteria in macrophages from HIV‐positive patients but not in HIV‐negative controls, suggesting vitamin D may improve macrophage functions in HIV‐positive patients (Haug *et al*. 1998). Despite the significant body of evidence that has shown that vitamin D metabolites can modulate the immune response in HIV patients, it is unclear whether vitamin D supplementation can reduce morbidity and mortality in HIV‐positive patients.

The second main finding of this study is that the vitamin D status of cats with FIV is not lower than FIV‐negative ill cats which have been hospitalized with a range of conditions. It is unclear if low serum 25(OH)D concentrations are simply a non‐specific change which commonly occurs in ill cats or whether vitamin D status plays a physiological role in disease development and progression Potential causes for low vitamin D in illness in cats include; reduced dietary intake and effects of drugs or haemodilution from intravenous fluids. Some medications such as glucocorticoids can infulence vitamin D metabolism in humans. it is unclear whether many commonly used drugs can influence vitamin D metabolism in cats. For example, it has previously been shown that glucocorticoids do not alter vitamin D metabolism in dogs (Kovalik *et al*. 2012a). In light of paucity of information on how drugs influence vitamin D homeostasis, it was elected to take an unbiased approach of not excluding any cases based on previous medical therapy , with the exception of corticosteroids as outline in the methods and materials. Further research is needed to conclude if these are relevant factors in feline medicine. In addition, some studies have suggested that vitamin D may be a negative acute phase reactant (Waldron *et al*. 2013).

A growing body of data in human medicine has shown that 25(OH)D concentrations are negatively associated with the all‐cause mortality and vitamin D supplementation may ameliorate morbidity (Amrein *et al*. 2014; Schottker *et al*. 2014). The presence of vitamin D receptors on immune cells combined with experimental evidence shows that vitamin D can markedly modulate the immune system may partly explain why vitamin D status appear to have such an important association with outcome in a number of diseases. Less is known about the relationship between vitamin D status and disease outcomes in cats although studies have documented low serum 25(OH)D levels in diseases including inflammatory bowel disease, gastrointestinal lymphoma and mycobacterial infection (Lalor *et al*. 2012, 2014). It has been demonstrated that low serum concentrations 25(OH)D are associated with an increased risk of 30 day mortality in sick cats (Titmarsh *et al*. 2015). In addition it has been shown that low vitamin D status is associated with a poorer response to prednisolone treatment for canine atopic skin disease (Kovalik *et al*. 2012b).

There are several limitations in this study. Firstly, signalment and clinical data were missing from the FIV‐infected cohort. Although this limits our ability to conclude whether reduced 25(OH)D concentrations are a direct consequence of FIV infection or due to co‐morbid disease, the results do indicate that cats with FIV infections are not any greater risk of having lower vitamin D status than FIV negative, hospitalized ill cats. The other limitation of the study is that different ELSIA tests were used to assess FIV status. In general, in house tests for FIV have a similar specificity and sensitivity, (Hartmann *et al*. 2007) making it unlikely that the use of different kits would adversely affect the validity of our results. Lastly, both very old and very young cats were enrolled in the study and age may vary being these groups. Currently, knowledge regarding the effects of age on vitamin D status in cats is limited to studies indicating that 25(OH)D increase over time in growing cats (Pineda *et al*. 2013).

Prospective studies investigating the effects of vitamin D status on susceptibility to illness and treatment outcomes are warranted to investigate the potential importance between the association of low vitamin D status and health outcomes. In addition, future studies which investigate serum vitamin D concentrations in clinically well and clinically ill FIV‐infected cats would allow the association of secondary illnesses on vitamin D status to be assessed. Future studies investigating the effect of dietary composition or appetite on vitamin D status would also be valuable.

## Conclusion

In summary, our study has shown that FIV‐infected cats have a significantly lower vitamin D status than healthy control cats. The vitamin D status of FIV‐infected, hospitalized ill and healthy cats and the relationship between vitamin D status and long‐term prognosis in FIV‐infected cats are deserving of further study.

## Conflicts of interest

The authors disclose no conflict of interest

## References

[vms311-bib-0001] Amrein K. , Schnedl C. , Holl A. , Riedl R. , Christopher K.B. , Pachler C. *et al* (2014) Effect of high‐dose vitamin D3 on hospital length of stay in critically ill patients with vitamin D deficiency: the VITdAL‐ICU randomized clinical trial. JAMA 312, 1520–1530. 25268295 10.1001/jama.2014.13204

[vms311-bib-0002] Campbell G.R. & Spector S.A. (2011) Hormonally active vitamin D3 (1*α*,25‐dihydroxycholecalciferol) triggers autophagy in human macrophages that inhibits HIV‐1 infection. Journal of Biological Chemistry 286, 18890–18902. 21454634 10.1074/jbc.M110.206110 PMC3099705

[vms311-bib-0003] Dow S.W. , Poss M.L. & Hoover E.A. (1990) Feline immunodeficiency virus: a neurotropic lentivirus. Journal of Acquired Immune Deficiency Syndromes 3, 658–668. 2161920

[vms311-bib-0500] Dunham S. , & Jarrett O. (2006) FIV as a model for AIDS vaccine studies. In: Friedman H., Specter S., Bendinelli M., eds. In vivo models of HIV disease and control US: Springer, pp. 293–332.

[vms311-bib-0004] English R.V. , Johnson C.M. , Gebhard D.H. & Tompkins M.B. (1993) In vivo lymphocyte tropism of feline immunodeficiency virus. Journal of Virology 67, 5175–5186. 7688819 10.1128/jvi.67.9.5175-5186.1993 PMC237915

[vms311-bib-0005] Hahn T.J. , Halstead L.R. & Baran D.L.T. (1981) Effects of short term glucocorticoid administration on intestinal calcium absorption and circulating vitamin D metabolite concentrations in man. Journal of Clinical Endocrinology and Metabolism 52, 111–115. 6969728 10.1210/jcem-52-1-111

[vms311-bib-0006] Hartmann K. (2011) Clinical aspects of feline immunodeficiency and feline leukemia virus infection. Veterinary Immunology and Immunopathology 143, 190–201. 21807418 10.1016/j.vetimm.2011.06.003 PMC7132395

[vms311-bib-0007] Hartmann K. , Griessmayr P. , Schulz B. , Greene C.E. , Vidyashankar A.N. , Jarrett O. *et al* (2007) Quality of different in‐clinic test systems for feline immunodeficiency virus and feline leukaemia virus infection. Journal of Feline Medicine and Surgery 9, 439–445. 17604205 10.1016/j.jfms.2007.04.003 PMC10911499

[vms311-bib-0008] Haug C. , Müller F. , Aukrust P. & Frøland S.S. (1994) Subnormal serum concentration of 1,25‐vitamin D in human immunodeficiency virus infection: correlation with degree of immune deficiency and survival. Journal of Infectious Diseases 169, 889–893. 7907645 10.1093/infdis/169.4.889

[vms311-bib-0009] Haug C.J. , Müller F. , Rollag H. , Aukrust P. , Degré M. , FrøLand S.S. (1996) The effect of 1,25‐vitamin D3 on maturation of monocytes from HIV‐infected patients varies with degree of immunodeficiency. Acta Pathologica, Microbiologica, et Immunologica Scandinavica 104, 539–548. 10.1111/j.1699-0463.1996.tb04909.x 8920807

[vms311-bib-0010] Haug C.J. , Müller F. , Aukrust P. & Frøland S.S. (1998) Different effect of 1,25‐dihydroxyvitamin D3 on replication of *Mycobacterium avium* in monocyte‐derived macrophages from human immunodeficiency virus‐infected subjects and healthy controls. Immunology Letters 63, 107–112. 9761372 10.1016/s0165-2478(98)00065-0

[vms311-bib-0011] Holick Michael F. (2009) Vitamin D status: measurement, interpretation, and clinical application. Annals of epidemiology 19.2, 73–78. 18329892 10.1016/j.annepidem.2007.12.001 PMC2665033

[vms311-bib-0012] Beard, Jeremy A. , Allison Bearden and Rob Striker. (2011) Review: vitamin D and the anti‐viral state. Journal of Clinical Virology 50, 194–200. 21242105 10.1016/j.jcv.2010.12.006 PMC3308600

[vms311-bib-0013] Kim J.H. , Gandhi V. , Psevdos G. Jr , Espinoza F. , Park J. & Sharp V. (2012) Evaluation of vitamin D levels among HIV‐infected patients in New York City. AIDS Research and Human Retroviruses 28, 235–241. 21644847 10.1089/AID.2011.0040

[vms311-bib-0014] Kovalik M. , Thoday K.L. , Evans H. , Berry J. , van den Broek A.H.M. & Mellanby R.J. (2012a) Short‐term prednisolone therapy has minimal impact on calcium metabolism in dogs with atopic dermatitis. The Veterinary Journal 193, 439–442. 22277720 10.1016/j.tvjl.2011.12.003

[vms311-bib-0015] Kovalik M. , Thoday K.L. , Berry J. , van den Broek A.H.M. & Mellanby R.J. (2012b) Prednisolone therapy for atopic dermatitis is less effective in dogs with lower pretreatment serum 25‐hydroxyvitamin D concentrations. Veterinary Dermatology 23, 125.e28. doi: 10.1111/j.1365‐3164.2011.01022.x 22141403 10.1111/j.1365-3164.2011.01022.x

[vms311-bib-0016] Lake J.E. & Adams J.S. (2011) Vitamin D in HIV‐infected patients. Current HIV/AIDS Reports 8, 133–141. 21647555 10.1007/s11904-011-0082-8 PMC3666828

[vms311-bib-0017] Lalor S.M. , Mellanby R.J. , Friend E.J. , Bowlt K.L. , Berry J. & Gunn‐Moore D. (2012) Domesticated cats with active mycobacteria infections have low serum vitamin D (25(OH)D) concentrations. Transboundary and Emerging Diseases 59, 279–281. 21999899 10.1111/j.1865-1682.2011.01265.x

[vms311-bib-0018] Lalor S. , Schwartz A.M. , Titmarsh H. , Reed N. , Tasker S. , Boland L. *et al* (2014) Cats with inflammatory bowel disease and intestinal small cell lymphoma have low serum concentrations of 25‐hydroxyvitamin D. Journal of Veterinary Internal Medicine 28, 351–355. 24433362 10.1111/jvim.12294 PMC4858012

[vms311-bib-0019] Mawer E.B. , Berry J.L. , Cundall J.P. , Still P.E. & White A. (1990) A sensitive radioimmunoassay using a monoclonal antibody that is equipotent for ercalcitriol and calcitriol (1,25‐dihydroxy vitamin D2 and D3). Clinica Chimica Acta 190, 199–209. 10.1016/0009-8981(90)90174-q 2253400

[vms311-bib-0020] Mehta S. , Mugusi F.M. , Spiegelman D. , Villamor E. , Finkelstein J.L. , Hertzmark E. *et al* (2011) Vitamin D status and its association with morbidity including wasting and opportunistic illnesses in HIV‐infected women in Tanzania. AIDS Patient Care and STDS 25, 579–585. 21916603 10.1089/apc.2011.0182 PMC3183700

[vms311-bib-0021] Novotney C. , Housman J. , Davidson M.G. , Nasisse M.P. , Jeng C.‐R. , Davis W.C. *et al* (1990) Lymphocyte population changes in cats naturally infected with feline immunodeficiency virus. AIDS 4, 1213–1218. 1982410 10.1097/00002030-199012000-00005

[vms311-bib-0022] Pedersen N. , Yamamoto J.K. , Ishida T. & Hansen H. (1989) Feline immunodeficiency virus infection. Veterinary Immunology and Immunopathology 21, 111–129. 2549690 10.1016/0165-2427(89)90134-7

[vms311-bib-0023] Peterlin B.M. & Trono D. (2003) Hide, shield and strike back: how HIV‐infected cells avoid immune eradication. Nature Reviews Immunology 3, 97–107. 10.1038/nri998 12563294

[vms311-bib-0024] Pineda C. , Aguilera‐Tejero E. , Guerrero F. , Raya A.I. , Rodriguez M. & Lopez I. (2013) Mineral metabolism in growing cats: changes in the values of blood parameters with age. Journal of Feline Medicine and Surgery 15, 866–871. doi:10.1177/1098612X13478264. 23413272 10.1177/1098612X13478264 PMC11383153

[vms311-bib-0025] Prietl B. , Treiber G. , Pieber T.R. & Amrein K. (2013) Vitamin D and immune function. Nutrients 5, 2502–2521. 23857223 10.3390/nu5072502 PMC3738984

[vms311-bib-0026] Ramsey I.K. , Tebb A. , Harris E. , Evans H. & Herrtage M.E. (2005) Hyperparathyroidism in dogs with hyperadrenocorticism. Journal of Small Animal Practice 46, 531–536. 16300114 10.1111/j.1748-5827.2005.tb00282.x

[vms311-bib-0027] Schottker B. , Jorde R. , Peasey A. , Thorand B. , Jansen E.H. , Groot L. *et al* (2014) Vitamin D and mortality: meta‐analysis of individual participant data from a large consortium of cohort studies from Europe and the United States. British Medical Journal (Clinical Research Ed) 348, g3656. 10.1136/bmj.g3656 PMC4061380 24938302

[vms311-bib-0028] Titmarsh H. , Kilpatrick S. , Sinclair J. , Boag A. , Bode E.F. , Lalor S.M. *et al* (2015) Vitamin D status predicts 30 day mortality in hospitalised cats. PLoS One 10, e0125997. 25970442 10.1371/journal.pone.0125997 PMC4430519

[vms311-bib-0029] Tompkins M.B. & Tompkins W.A. (2008) Lentivirus‐induced immune dysregulation. Veterinary Immunology and Immunopathology 123, 45–55. 18289702 10.1016/j.vetimm.2008.01.011 PMC2410212

[vms311-bib-0030] Vescini F. , Cozzi‐Lepri A. , Borderi M. , Re M.C. , Maggiolo F. , De Luca A. *et al* (2011) Prevalence of hypovitaminosis D and factors associated with vitamin D deficiency and morbidity among HIV‐infected patients enrolled in a large Italian cohort. Journal of Acquired Immune Deficiency Syndromes (1999) 58, 163–172. 21826011 10.1097/QAI.0b013e31822e57e9

[vms311-bib-0031] Viard J.P. , Souberbielle J.C. , Kirk O. , Reekie J. , Knysz B. , Losso M. *et al* (2011) Vitamin D and clinical disease progression in HIV infection: results from the EuroSIDA study. Aids 25.10, 1305–1315. 10.1097/QAD.0b013e328347f6f7 21522006

[vms311-bib-0032] Villamor E. (2006) A potential role for vitamin D on HIV infection? Nutrition Reviews 64, 226–233. 16770943 10.1301/nr.2006.may.226-233

[vms311-bib-0033] Waldron J.L. , Ashby H.L. , Cornes M.P. , Bechervaise J. , Razavi C. , Thomas O.L. *et al* (2013) Vitamin D: a negative acute phase reactant. Journal of Clinical Pathology 66, 620–622. 23454726 10.1136/jclinpath-2012-201301

[vms311-bib-0034] Yamamoto J.K. , Sparger E. , Ho E. , Andersen P.R. , O'connor T. , Mandell C. *et al* (1988) Pathogenesis of experimentally induced feline immunodeficiency virus infection in cats. American Journal of Veterinary Research 49, 1246–1258. 2459996

