# Vitamin D status in cats with feline immunodeficiency virus

**DOI:** 10.1002/vms3.27

**Published:** 2016-01-22

**Authors:** Helen F. Titmarsh, Stephanie M. Lalor, Severine Tasker, Emily N. Barker, Jacqueline Berry, Danielle Gunn‐Moore, Richard J. Mellanby

In the originally published version of this article by Titmarsh *et al*. 2015, the last name of one of the authors was misspelled. The correct spelling is now reflected here as Gunn‐Moore instead of Gunn‐More.

We apologize for the error.
